# Detection and Classification of Multirotor Drones in Radar Sensor Networks: A Review

**DOI:** 10.3390/s20154172

**Published:** 2020-07-27

**Authors:** Angelo Coluccia, Gianluca Parisi, Alessio Fascista

**Affiliations:** Dipartimento di Ingegneria dell’Innovazione, University of Salento, via Monteroni, 73100 Lecce, Italy; gianluca.parisi@studenti.unisalento.it (G.P.); alessio.fascista@unisalento.it (A.F.)

**Keywords:** multi-rotor drones, UAV, detection, classification, radar

## Abstract

Thanks to recent technological advances, a new generation of low-cost, small, unmanned aerial vehicles (UAVs) is available. Small UAVs, often called drones, are enabling unprecedented applications but, at the same time, new threats are arising linked to their possible misuse (e.g., drug smuggling, terrorist attacks, espionage). In this paper, the main challenges related to the problem of drone identification are discussed, which include detection, possible verification, and classification. An overview of the most relevant technologies is provided, which in modern surveillance systems are composed into a network of spatially-distributed sensors to ensure full coverage of the monitored area. More specifically, the main focus is on the frequency modulated continuous wave (FMCW) radar sensor, which is a key technology also due to its low cost and capability to work at relatively long distances, as well as strong robustness to illumination and weather conditions. This paper provides a review of the existing literature on the most promising approaches adopted in the different phases of the identification process, i.e., detection of the possible presence of drones, target verification, and classification.

## 1. Introduction

Unmanned Aerial Vehicles (UAVs), more commonly known as *drones*, have been recognized as one of the revolutionary advances in the recent technological evolution, and are now experiencing new applications within the information and communication technology (ICT) ecosystem [1]. The origin of UAVs traces back to the military field, for operating in hostile or human-denied areas. Over the past decade, UAVs have become increasingly more attractive for a plethora of applications also in civilian and commercial domains, including surveillance [2], search and rescue [3], traffic and weather monitoring [4], precision agriculture [5], aerial imaging [6], and others [7].

The progress in the market of device miniaturization and the consequent reduction of the production costs made such technologies accessible also to the general public. Nowadays, small UAVs (quadcopters or different variants of rotorcrafts with a weight below 20 kg) can be easily purchased on the Internet or even built from scratch using low-cost commercial off-the-shelf (COTS) development kits for a few hundreds of dollars. The wide diffusion of small commercial UAVs opened up a new set of opportunities, but at the same time poses a number of threats in terms of safety, privacy and security [8]. Indeed, despite their small size, UAVs can carry payloads up to one kilogram or more, and are equipped with cameras and wireless communication systems. Unfortunately, these characteristics make them perfect platforms for performing nefarious activities. Several cases of different kinds have been already reported in many countries. For instance, criminal organizations in Canada and Australia exploited UAVs to smuggle drugs or other illicit materials into prisons or across the borders [9,10]. In 2015, a very small UAV was used for espionage at Kuala Lumpur International Airport [11]. At the Black Hat international conference, a group of researchers demonstrated that UAVs can be equipped with modified operative systems and used to steal sensitive data from mobile phones in crowded environments by simply leveraging the automatic search of Wi-Fi networks [12]. More generally, amateur drone pilots are nowadays widespread all over the world, concurring to make very difficult to discriminate situations in which UAVs are used for recreational activities from those in which such devices are maliciously used to harm the public safety.

The exponential growth of such flying objects and the several issues related to their possible misuse is mandating the need for increasingly more sophisticated drone identification systems. There is indeed a gap in current surveillance systems: the typical small size and the ability to perform very fast maneuvers make UAVs a category of targets much more difficult to reveal compared to traditional aircrafts [13]. More precisely, the following main aspects should be carefully taken into account:

- UAVs can be correctly identified only at very short distances. Effective surveillance systems must be able to react and take the appropriate countermeasures promptly, also in adverse operating conditions such as low visibility, propagation environments full of obstacles, etc.
- Major security threats can arise from UAVs approaching in swarms. The adopted technologies should be able to detect multiple targets simultaneously and to track their trajectories in real-time.
- UAVs cannot be easily distinguished from other small flying objects such as birds. Advanced signal processing algorithms are then needed in order to lower the probability of false alarm and increase the correct detection rate.

**Figure 1:**
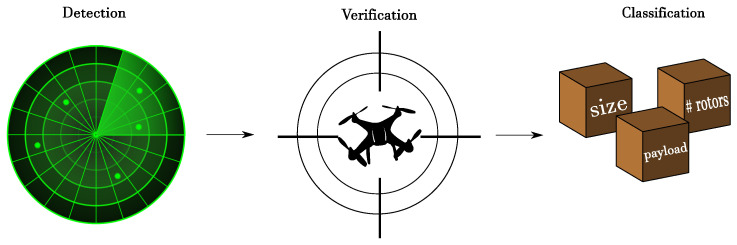
Target identification process.

The problem of target *identification* can be recast as a (complex) macro-task that includes three different phases. The first one, called *detection*, consists of using the statistical theory of hypothesis testing to make a decision about the possible presence of a target. Once a detection has been triggered, a *verification* phase may follow to verify whether the target is actually present or not. This phase is important to reduce the number of false alarms that can be erroneously produced in the first phase, and can be approached in many different ways, also using external information, complementary systems, or even manual intervention. If the detection is confirmed, a final *classification* step assigns the target to a specific category based on some distinguishing attributes (e.g., size, number of rotors, max payload, …). The entire process is depicted in Figure 1.

**Figure 2:**
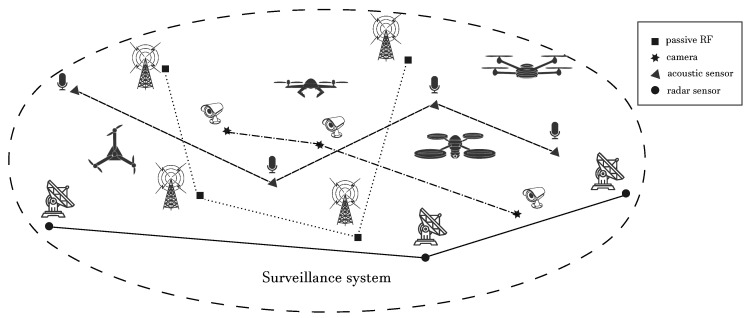
Heterogeneous drone surveillance system.

The conceptual scheme of a typical distributed surveillance system for drone identification is depicted in Figure 2; several detection and classification technologies that are currently under investigation are shown, including acoustic sensors, (multi-spectral) cameras with video analytics, LIDAR (light detection and ranging), and radio frequency (RF) based detection sensors, either passive or active. In fact, although significant improvements have been made regarding the use of such methods for drone identification, it clearly emerged that the requirements discussed above cannot be jointly satisfied using a single specific technology. To tackle such complex tasks, the research community steered its attention towards novel integrated and hybrid solutions that approach the problem of drone identification using a network of spatially-distributed heterogeneous sensors (e.g., cameras, radars, microphones), which are used to collect and process different types of data about the target. The main idea consists of exploiting the information gathered from the heterogeneous sensors in the network in a complementary way, with the aim of providing a holistic surveillance system able to promptly detect the presence of UAVs and track their trajectories in real-time, while coping at the same time with the different operating conditions at hand (e.g., long range, low visibility,…). Systems implementing such an integrated approach have been recently investigated also in Horizon2020 European research projects *SafeShore* (https://safeshore.eu) and *Aladdin* (https://aladdin2020.eu). In the former, the main goal was the surveillance of coastal borders to detect small UAVs and boats, which can potentially carry explosives or can be used for smuggling [14]; the involved technologies are 3D LIDAR sensors, passive radio detectors, video analytics, and passive acoustic sensors. Within the same project, a “drone-vs-bird detection” grand challenge has been launched (the challenge is at its third edition, ref. https://wosdetc2020.wordpress.com/drone-vs-bird-detection-challenge/), with the aim of addressing the technical issue of discriminating between drones and birds [15,16]. Similarly, the Aladdin project, still ongoing, has the goal of developing an innovative drone detection and neutralization system based on the adoption of different technologies including acoustic sensors, radar, and cameras. As discussed, indeed, to deal with the particularly challenging category of small-drone targets over an even moderately large area, a distributed sensor network is needed including various technologies. All these technologies have pros and cons thus, if individually considered, can be effective only under specific conditions; the network becomes thus of fundamental importance in order to correctly execute the three phases constituting the identification process.

The vast majority of literature on the topic of drone identification focuses on image processing and video analytics based on the training of classifiers, namely machine learning algorithms, fed by video sequences collected from different types of cameras. The goal is to extract some features for different types of classifications: drone category (fixed wing, single-rotor, multi-rotor); distinction between drones and birds, which are the most similar targets for size and radar cross section (RCS); evaluation of the presence of any payload that affects the RCS of the entire target [17,18]. The main limitation of this technology for primary detection is the size of such targets, which can be easily confused with the background or indistinguishable from birds. Moreover, cameras are severely affected by environmental conditions, for instance low ambient light, variable illumination and shadows, or even simple occlusion of the lens that turns the system completely inoperative; for night time and generally dark areas infrared cameras are also required, which have usually lower resolution and range, and higher cost. Due to all these considerations, video-based approaches are very useful in good weather conditions and at short distances, especially for the verification and classification phases after having already declared a detection. The reader is referred to [19] for a recent comprehensive review of the existing video-based techniques for drone detection. Less surveyed is instead the literature on radar techniques. In this respect, an original contribution of this paper is the focus on the review of radar-based detection and classification work.

Before delving into the details of radio frequency approaches, in particular based on active sensors (radar), it is worth mentioning that acoustic sensors have been also considered as a viable technology to identify the presence of drones based on the application of statistical array processing techniques. More specifically, the use of audio sensors is meant to grasp the sound of both motors and fast rotating propellers (for a single/multi-rotor drone) to distinguish these kinds of targets from others, especially birds. Due to the size of the targets and ambient noise, the sensitive range of these sensors, and consequently their detection capability, is very limited. However, they might still be useful for the second phase of identification, i.e., verification: in fact, once the detection of a generic target has been declared, it is possible to extract a sound signature to either confirm the acquired target or declare a false alarm [20,21]. The reader is referred to [22,23] for a more deep discussion regarding audio-based drone detection technologies.

RF signals are robust to weather and illumination conditions, and can provide medium to long range coverage. They are a suitable tool to handle the primary detection phase, which triggers the entire identification process. The most important device for *active* RF-based drone detection is the radar sensor, but *passive* technologies are also relevant. In particular, the use of spectrum sensing can be a prominent solution to detect a drone when uplink/downlink transmissions exist between the drone and its controller. This may also enable localization of the remote pilot, which is important from a liability point of view. However, there are two limitations in this approach, related to the portion of the spectrum on which communications between the controller and the drone take place, and to the possibility of autonomous (GPS-based) guidance mode. Indeed, drone communications typically use the industrial, scientific and medical (ISM) frequency spectrum where many other systems (including Wi-Fi and some fixed wireless access technologies) are found; this means the band of interest is crowded, increasing the risk of false alarms. To cope with this issue, several papers have addressed the subject of network traffic analysis, see, for example, [24,25,26,27,28,29]. Nonetheless, passive detection is completely ineffective in the case of drones flying in fully autonomous mode, which can be especially the case of security threats. Laser scanners (LIDAR) are also considered for active detection in environments where radars cannot be used. In this case, backscattering of laser light is exploited, which however is sensitive to bad visibility due to weather, smog, or direct sunlight. In normal weather conditions, LIDARs can be very effective for drone detection and basic classification, namely based on target size, which can occupy several cells as a consequence of the fine angular resolution of lasers (very narrow beam). However, targets with similar size, in particular drones and birds, are indistinguishable. LIDARs can be thus considered a complementary technology with respect to RF sensors.

Radar sensors remain an important component of drone surveillance systems. As known, this approach is essentially based on the electromagnetic principle of backscattering, which occurs when an object is illuminated by the radar beam. The main difficulty for the case of small drones is that the probability of detection is highly dependent on the radar cross section of the targets which, as already mentioned, is rather small for drones that are mostly void and made of plastic. For this reason, new radar setups have been developed trying to exploit the backscatter from rotating parts like propellers and rotors to evaluate micro-Doppler signatures. Especially for the third phase of classification, micro-Doppler analysis can provide useful information regarding the number of propellers and rotors (single/multi-rotor). Also, in case of a false alarm due to a bird, the time–frequency analysis may enable accurate discrimination. The goal of this paper is to review the existing work on drone identification based on radar sensors. In addition to the classification task, the focus is put on the first phase of the identification process, i.e., the detection phase. This has been much less explored compared to the classification phase where, in most cases, the presence of a target is assumed upfront. A comparison among the different technologies adopted in the literature is summarized in Table 1, with specific focus on the type of approaches used for identifying the presence of drones as well as on the main pros and cons of each technology.

## 2. Basic Theory for Radar Signal Processing

Before proceeding further, a brief review of some basic concepts of radar signal processing is provided; for more details, the reader is referred to [30,31,32].

### 2.1. Radar Sensor

Due to the small size of drones, given the limitation in bandwidth and power, and, not least, the expensive cost, pulsed surveillance radars cannot be often adopted, especially when a network of radars must be deployed to guarantee a full-coverage of the monitored area, as is the case in drone identification. *Frequency modulated continuous wave (FMCW)* and *continuous wave (CW)* radars currently represent the most appealing and cost-effective solution to tackle this problem.

**Figure 3:**
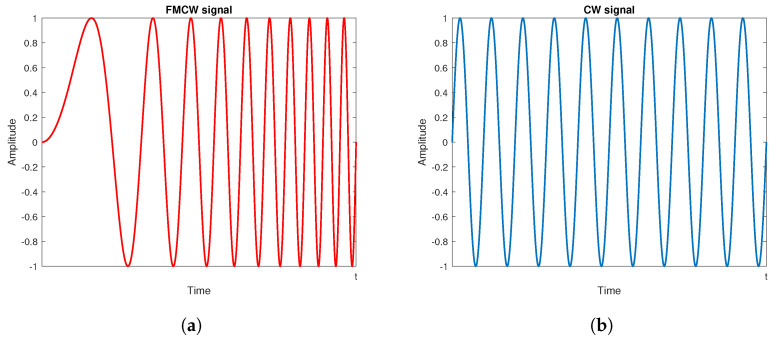
(**a**) Frequency modulated continuous wave (FMCW) and (**b**) continuous wave (CW) signals.

**Figure 4:**
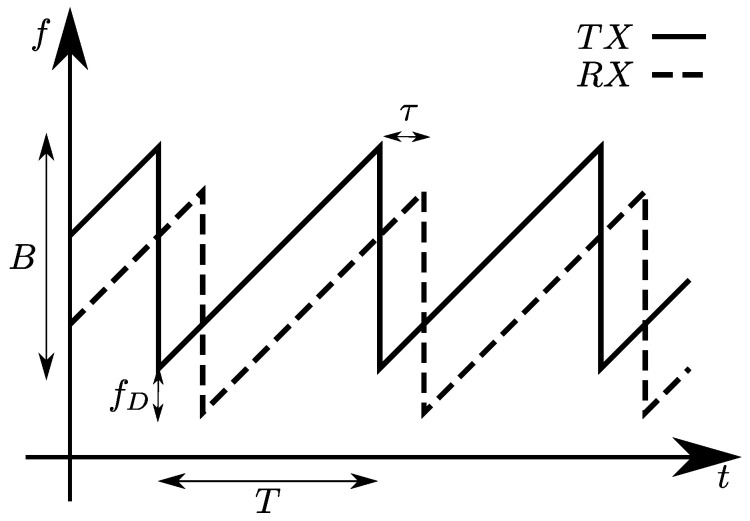
FMCW transmitted and received signals.

**Figure 5:**
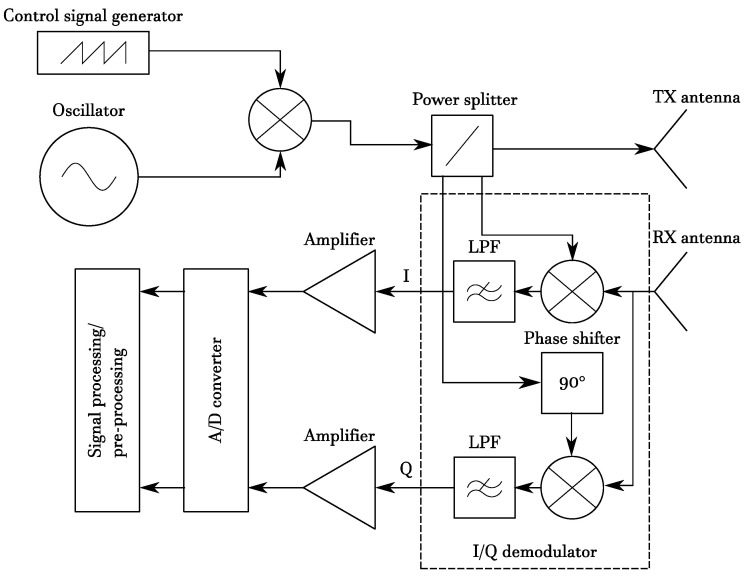
FMCW radar architecture.

An FMCW signal, also known as linear FMCW (LFMCW) or linear frequency modulated (LFM) signal, consists of a linearly modulated continuous wave radio energy transmitted in a desired direction. These kinds of signals are often referred to as *chirps* and differ from CW because, in the latter, the operating frequency is not varied during the transmission, as shown in Figure 3a,b. Radars using such waveforms became very popular especially in the automotive field, due to the low cost of hardware components, and thanks to their ability to provide both range and Doppler information. The FMCW transmitted and received signals are schematically depicted in Figure 4; from these signals, it is possible to extract delay τ and phase φ information, which are useful to obtain distance and velocity information of one or more targets simultaneously. The basic processing of the received signal is performed through I/Q demodulation (as reported in Figure 5), which provides in-phase and quadrature-phase components of the complex baseband signal, called *beat signal* or Intermediate Frequency (IF) signal. The down-conversion is introduced to considerably simplify the realization of the processing circuits, allowing the circuit to work at much lower frequency compared to the transmitted signal. The substantial difference between FMCW and CW processing chains, see Figure 5, is only in the control signal generator, which provides the reference signal (in the CW case it is a constant one). Combining this signal with a voltage controlled oscillator (VCO) produces the resulting RF signal that will be transmitted by the radar.

Mathematically speaking, the transmitted FMCW signal can be expressed as

(1) $$s_{TX}\left(t\right)=A_{TX}\cos\left(2\pi f_{c}t+\pi St^{2}+\varphi_{TX}\right),$$

where $A_{TX}$ is the amplitude, $\varphi_{TX}$ is the phase shift, S=B/T is the slope of the chirp and $f_{c}$ is the carrier frequency. If in t=0 the target is at a distance *d* from the radar, then the received signal will be a delayed and attenuated version of the transmitted signal, i.e.,

(2) $$s_{RX}\left(t\right)=A_{RX}\cos\left(2\pi f_{c}\left(t-\tau \right)+\pi S{\left(t-\tau \right)}^{2}+\varphi_{RX}\right)+n\left(t\right),$$

where n(t) is the noise component and τ≈2d/c (with *c* the speed of light) is the delay, i.e., the round trip time. In-phase and quadrature-phase components of the IF (beat) signal can be expressed as

(3) $$\begin{array}{cc} IF(t) & =IF_{I}\left(t\right)+jIF_{Q}\left(t\right)\\ & =A_{IF}e^{j(2\pi f_{c}\tau +2\pi S\tau t-\pi S\tau^{2}-\Delta \varphi )}+n_{I}\left(t\right)+jn_{Q}\left(t\right)\\ & =A_{IF}e^{j\Psi (t)}+n_{I}\left(t\right)+jn_{Q}\left(t\right), \end{array}$$

where Ψ(t) denotes the phase of the beat signal. The previous expressions are still valid for the CW case, the only difference being the slope (chirp rate) *S* which for CW is equal to zero. Then, the instantaneous frequency related to the signal backscattered from the target is the derivative of Ψ(t), i.e.,

(4) $$f_{IF}=\frac{1}{2\pi }\frac{d\Psi (t)}{dt}=S\tau .$$

Recalling the definition of τ and using Equation (4), it is easy to estimate the range of the target as

(5) $$\hat{d}=\frac{c}{2S}f_{IF}.$$

The above range estimation holds only for stationary objects. To obtain *range* and *velocity* estimates for a moving object such as a drone, it is necessary to consider the variation in distance as a function of time, producing in turn a time-varying τ, i.e.,

(6) $$\tau \left(t\right)=\frac{2d}{c}+\frac{2v_{r}t}{c}\cos\left(\theta \right),$$

where $v_{r}=\frac{\lambda }{2}f_{D}$ is the radial velocity of the target, depending on the Doppler frequency $f_{D}$, and θ is the aspect angle between the radar line-of-sight (LOS) and the target trajectory.

### 2.2. Moving Target Indicator (MTI)

In most cases, target detection aims to reveal the presence of objects that are not persistent in the observed scenario, i.e., objects that can dynamically appear or disappear. The *moving target indicator (MTI)* is a radar operating mode that allows the system to discriminate a target against both clutter and stationary objects in the monitored area. For this purpose, the Doppler effect principle plays a significant role, since stationary targets do not produce any Doppler frequency shift in the observed signals. In terms of the Doppler effect, the frequency shift of the backscattered signal will grow as the target is moving towards the LOS direction of the radar. Conversely, it will decrease if the target is moving away. Basically, this type of operating mode performs high-pass filtering, thus eliminating the low-frequency components associated with stationary targets. It is worth noting that this processing only provides feedback on the possible presence or absence of dynamic targets without specifying the number of them or evaluating their relative Doppler shifts. However, it still represents a valid support tool also because of its simple implementation. MTI filters are typically low-order, simple finite impulse response (FIR) designs, also known as tapped delay line. A prominent example is the two-pulse canceller digital filter, the input data of which are a sequence of baseband complex (I/Q) data samples from the same range bin over two successive pulses, forming a discrete-time sequence. The discrete-time transfer function of this filter is simply $H\left(z\right)=1-z^{-1}$. The frequency response as a function of the Doppler frequency $f_{D}$ is obtained by setting $z=e^{j2\pi f_{D}T}$, i.e.,

(7) $$\begin{array}{cc} H(f_{D}) & =\left(1-z^{-1}\right){|}_{z=e^{j2\pi f_{D}T}}\\ \; & =1-e^{-j2\pi f_{D}T}\\ \; & =e^{-j\pi f_{D}T}\left(e^{j\pi f_{D}T}-e^{-j\pi f_{D}T}\right)\\ \; & =2je^{-j\pi f_{D}T}\sin\left(\pi f_{D}T\right). \end{array}$$

The idea of cascading two-pulse canceller sections to obtain higher-order filters can be extended to the *N*-pulse canceller, obtained by cascading N−1 two-pulse canceller sections. The more general transfer function of the *N*-pulse canceller is therefore

(8) $$H_{N}\left(z\right)={\left(1-z^{-1}\right)}^{N-1}.$$

More details about this technique can be found in [30,33,34,35,36].

### 2.3. Features Extraction Techniques

There exists many different time–frequency analysis tools that can be leveraged to extract useful features for verifying and classifying a possible target. The most popular is the *Short Time Fourier Transform (STFT)*, which consist in a Fourier Transform applied on small portions of the received signal. There are numerous alternatives, such as the cadence velocity diagram (CVD) or the Cepstogram, which are completely based on the first evaluation of the *spectrogram*, that is, the STFT square modulus; or tools such as the Wigner–Ville distribution which, through correlation techniques, is able to show how the signal energy is distributed jointly in time and frequency.

**Figure 6:**
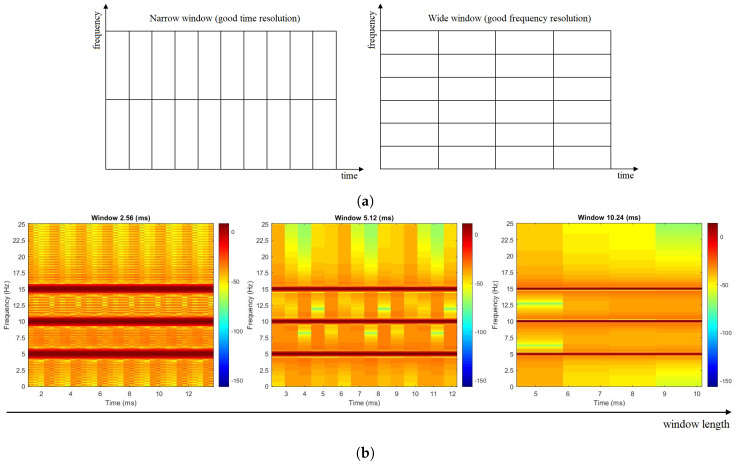
Short Time Fourier Transform (STFT) time–frequency resolution. (**a**) Narrow vs. Wide window (**b**) Example of time-frequency resolution for increasing window size.

In the following, the definition of STFT, which is the most commonly adopted technique, is reported:

(9) $$X\left[k\right]=\sum_{t=0}^{N-1}x\left[t\right]w\left[t-\tau \right]e^{-j2\pi k\frac{t}{N}}\;\mathrm{where}\;\begin{array}{c} \left\{k\in 0\cup N:k<N\right\}\\ \{\tau \in 0\cup N:\tau <N-M\}, \end{array}$$

where *k* and *t* are frequency and time, respectively, x[t] is the signal of interest, w[n] is a window function of length *M*, and *N* is the number of samples. The result of the STFT is generally a complex vector described by amplitude and phase, which are both time–frequency dependent. As mentioned, a typical mechanism to evaluate the micro-Doppler signature is the spectrogram. A possible limitation of the STFT is the fixed resolution. Indeed, the choice of the windowing function directly affects the representation of the signal: a narrow window gives good time resolution (the time at which frequencies change) but poor frequency resolution (Figure 6a left), while conversely a wide window gives better frequency resolution (frequency components close together can be separated) but poor time resolution (Figure 6a right). The trade-off between time and frequency resolution can be easily explained by recalling the Nyquist criterion for sampling. Since each frequency component is spaced by $f_{s}/M$, where $f_{s}$ is the sampling frequency, to effectively improve the frequency resolution one could decrease $f_{s}$ while keeping *M* constant; however, this will in turn reduce the number of samples per time, producing an increase of the window size. Alternatively, the frequency resolution can be improved by increasing *M*. In both cases, there is a reduction of the time resolution. The same type of reasoning applies to the temporal resolution in cases of narrow windows. For the sake of clarity, a concrete example considering a signal composed by three different frequency components (5, 10, and 15 Hz) for different window sizes is reported in Figure 6b.

### 2.4. Empirical Mode Decomposition (EMD)

*Empirical Mode Decomposition (EMD)* is an adaptive time–frequency technique that decomposes a signal into *intrinsic mode functions (IMF)* and a residue. The decomposition is based on the time-scale of the oscillations to obtain instantaneous frequency data. The first IMFs are those containing the highest frequency components, while the last ones have the lowest oscillating content. To be well-defined, each IMF must satisfy the following two conditions:

- The number of local extrema differs from the number of zero-crossings at most by one;
- The average of the envelope shall be zero.

**Figure 7:**
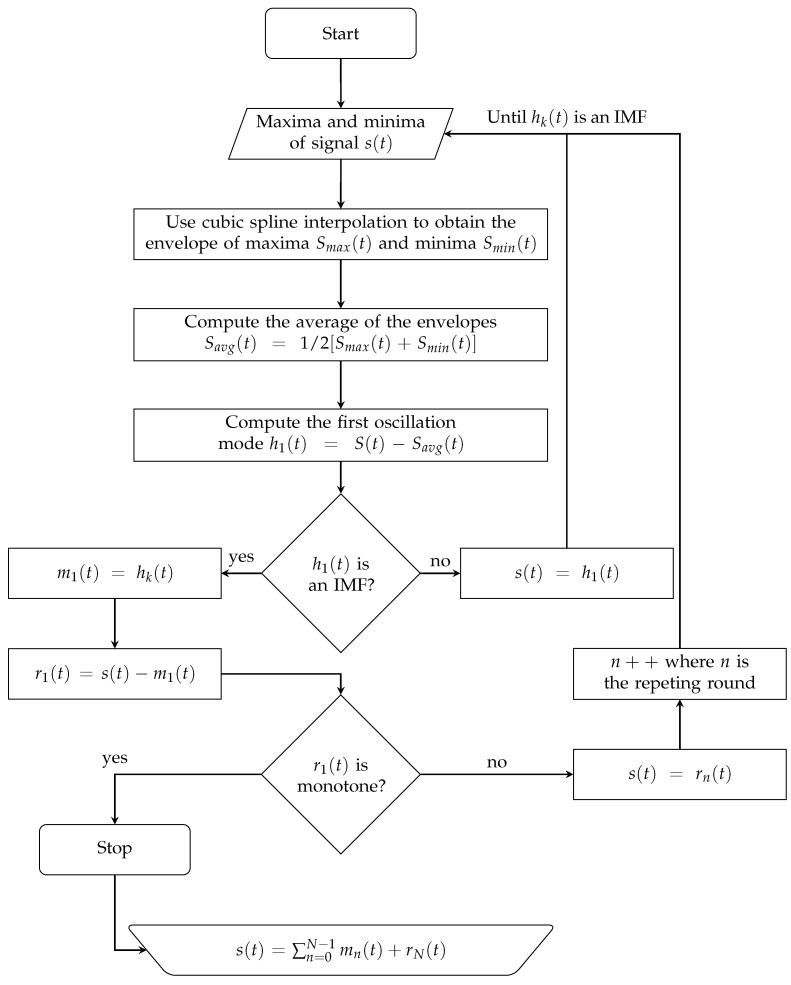
Flowchart of Empirical Mode Decomposition (EMD) algorithm.

Another interesting feature of the IMFs is the orthogonality between each of them, which represents a useful property for many applications. For instance, it is possible to perform signal filtering by selectively picking or adding some of the IMF together, while omitting the others. Denoting by s(t) the initial signal, the EMD algorithm basically consists of applying different shifting operations to that signal. The flowchart of such an approach is reported in Figure 7, more details can be found in [37].

### 2.5. Hardware Limitations and I/Q Imbalance

The most common problems that can be immediately faced when using a low-cost radar are the I/Q imbalance and the imperfections of the available hardware.

A first spurious effect that could impact the acquired data is the unexpected distortion of the signal after mixing. More specifically, this problem is caused by the local oscillator, which, instead of producing a pure LFM signal, generates a distorted version of the desired waveform. To overcome such a drawback, a preliminary calibration step should be performed so as to compensate for these potential deviations by trying to estimate the trend and subtracting them in order to clean up the signal. This type of effect, if untreated, could significantly affect the signal-to-noise ratio (SNR), resulting in poor estimation performances. This limitation is intrinsically hardware and can be partially overcome only by resorting to more accurate (hence more expensive) oscillators, which however cannot be easily found in common low-cost radars. Such effect has been analyzed, for instance, in [38].

Another important non-ideality that must be taken into account is the *I/Q imbalance*. As investigated in [38,39,40,41,42,43], this issue is caused by gain and phase errors that occur in the acquisition of the I and Q components, resulting in additional (undesired) sine waves. This effect leads to image responses at frequencies that are the negative with respect to the actual ones, and to correlated baseband noise.

## 3. Literature on Drone Detection

This section provides a review of the most relevant approaches for the small-drone detection task, including also some papers that are more focused on classification but use processing and techniques typical of radar detection. Beforehand, a basic background on radar detection and false alarm rate control is provided.

### 3.1. Constant False Alarm Rate (CFAR)

The simplest strategy that can be adopted in a typical detection problem is based on quantifying the energy carried by the return signal (or backscatter), namely, to use an *Energy Detector*. Although conceptually simple, this approach has an intrinsic weakness in the need to precisely know the disturbance level due to interference, clutter, and thermal noise, in order to properly set the decision threshold. Even small errors in the chosen value can lead to numerous false alarms or loss of detection (miss). In this perspective, approaches based on the estimation of the interfering contribution have been developed in order to adapt the threshold according to the data at hand.

*Constant False Alarm Rate (CFAR)* techniques are aimed at keeping constant a certain desired *Probability of False Alarm (PFA)* [44,45]. In general, CFAR detectors estimate statistics of the disturbance from radar returns and tune the detector threshold to guarantee the above-mentioned condition [46]. This threshold is expressed, in general, as the product of two terms $T=\alpha \hat{g}$, where $\hat{g}$ is the estimate of the interference statistic and α is the CFAR constant expressed as a function of the desired PFA.

**Figure 8:**
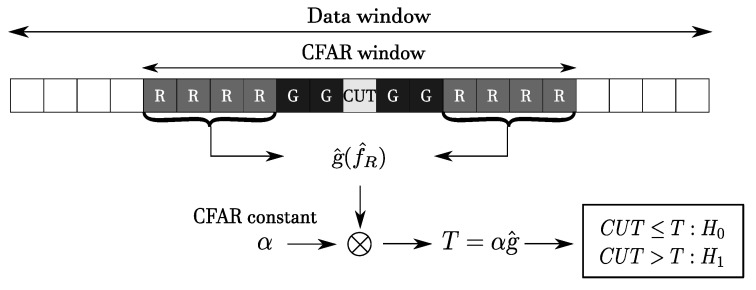
Basic monodimensional Constant False Alarm Rate (CFAR) architecture.

**Figure 9:**
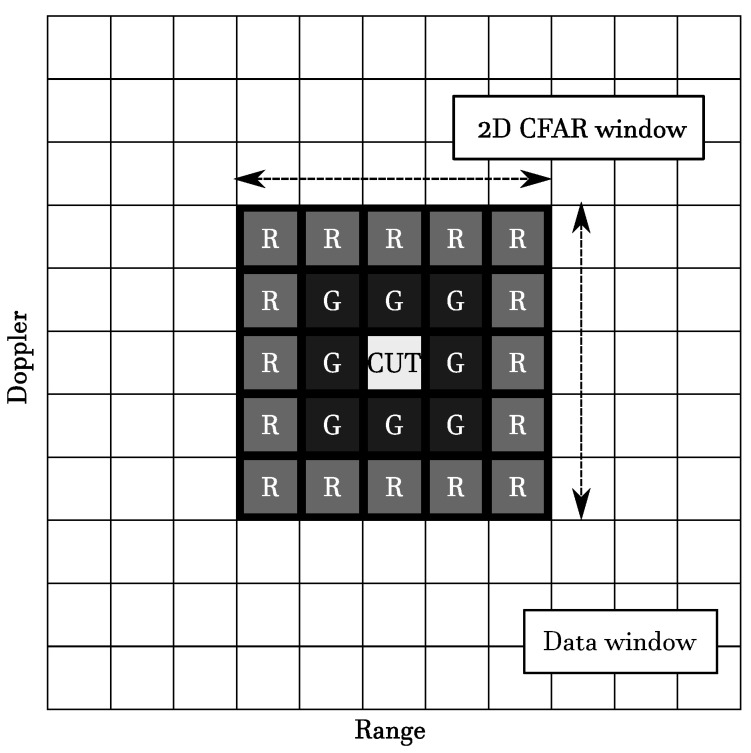
2D CFAR window.

The basic architecture for a CFAR detector is reported in Figure 8. The cells labeled as *G* are *guard cells*, and are considered or not according to the type of CFAR approach. Usually, they are introduced when it is expected that cells close to the *cell under test* (*CUT*) might be contaminated by returns, hence they are excluded from the estimation of the disturbance to avoid bias. The cells labeled as *R* are the *reference cells* used to construct the estimate of the interference statistic. The statistic computed on the CUT is compared to the CFAR threshold to determine the presence or absence of a target. All these quantities constitute the *CFAR window*, which is moved along the data dimension one cell at a time (and for each shift a detection decision is made regarding the measurement in the CUT). The CFAR window may also be multidimensional; for example, Figure 9 represents a 2D CFAR window.

The *Cell Averaging CFAR (CA-CFAR)* is the simplest version of a CFAR-based detector and is designed to operate in a quite favorable interference/target environment (homogeneous). An environment is defined homogeneous if the interference statistic is independent and identically distributed among all the cells of the CFAR window, and if the CUT contribution does not effect the reference window. If these conditions are not met, the environment is heterogeneous. In many scenarios, it is not realistic to expect only one target in the scenario; this might cause possible target returns in the reference window. Similarly, there might be a large target that exceeds the size of the CUT and thus contaminates the reference window [47]. These situations cause the erroneous introduction of a bias in the threshold and give rise to the so-called target *masking*: specifically, the first type is referred to as mutual masking and the second one as self-masking. A possible solution to the above-mentioned issues is the *Order Statistics CFAR (OS-CFAR)* [48,49]. In this approach, the statistics of each of the *R*-cells composing the CFAR reference window are ordered, and the *k*th one is selected as the CFAR statistic. This approach is thus capable of rejecting nR−k unwanted targets, where nR is the total number of cells in the reference window.

There also exist extensions and other versions of CFAR detectors based on these two discussed approaches. In [50] the Greatest of CA-CFAR technique is proposed, which reduces the clutter edge effect by using as the CFAR statistic the greatest of the samples mean for each reference window surrounding the CUT; on the other hand, in [51], the Smallest of CA-CFAR deals with the reduction of mutual target masking by using as the CFAR statistic the smallest of the noise power estimates.

In [52,53] the Censored CFAR has been defined: it calculates the average power in the reference windows after having ordered the samples in ascending order and discarded a certain number $N_{C}$ of them, in particular the ones corresponding to the highest values and therefore possibly returns from other targets. The way to set $N_{C}$ is related to prior assumptions on the number of expected interferers in the reference windows. In [54] a more general form of CS-CFAR called Trimmed Mean CFAR is adopted, which discards the $N_{L}$ largest and $N_{S}$ smallest samples.

Both OS and CS-CFAR are designed to deal with the target masking problem, by ranking the samples in the reference window and excluding some of them to compute the interference statistic. However, this approach could lead to a growth of false alarms due to clutter edge. To deal with this situation, many authors have investigated the possibility of using GO and SO techniques for such CFARs to improve robustness in presence of both multiple targets and *clutter boundaries*, i.e., significant localized changes in the interference power, as typically found in realistic scenarios [55,56,57,58]. Further details are beyond the scope of this work, but for the sake of completeness, Table 2 summarizes the above-mentioned CFAR detectors, for further details refer to [30].

### 3.2. Radar Detection Approaches

A few papers have addressed the detection problem as part of a more comprehensive classification problem. In [59] a micro-Doppler based system is proposed to detect and classify a drone without any CFAR statistic construction. A sensor network composed by low-cost radar sensors is used, which is capable of detecting and distinguishing drones against other typologies of targets very common in urban areas. In [60], a follow-up work of [61], a multistatic radar system (NetRAD) is used to collect data. The first step after the acquisition phase is a CFAR detector to identify the range cell of the drone and its state (flying/hovering). Finally, the classification of acquired targets is carried out.

The following works are conversely more specifically focused on the detection phase. In [62] an FMCW radar in K-band is distributed in two separate platforms and connected with optical fiber, in order to reduce the leakage coupling between transmit and receive antennas and improve the sensitivity of the system. By means of 2D Fast Fourier Transform (FFT), range and speed of the target are obtained, and thanks to the particular structure of the radar system, detection distances up to 500 m can be achieved.

In [63] two solutions based on FMCW radar operating in W-band are proposed for detecting small UAVs. The first is an 8 Hz rotating surveillance radar system supported with a camera; in this way for every second of monitoring, eight scans are obtained, which are then analyzed with an algorithm based on the CFAR approach. The second system is a multi-channel surveillance radar with four receiving antennas. This type of setup improves the spatial resolution in azimuth and elevation compared to the previous one. Experimental evaluations with both systems have shown that it is possible to detect a UAV up to about 100 m from the radar.

Authors in [64] addressed the detection problem using a multistatic radar composed of three nodes. A cell-averaging CFAR (CA-CFAR) approach was used for target detection. To discriminate UAVs from other targets, the micro-Doppler signature is analyzed by means of STFT. Furthermore, to track the target trajectory over time, an extended Kalman filter was used by merging the returns of each node in the multistatic system. Experimental evaluations showed that, in order to obtain an accurate tracking, a partial overlap is necessary between the beam of the transmitting antenna and at least one beam of the receiving antennas, otherwise outside this region the error increases and there may be track losses.

The issue of Range Migration (RM) and Doppler Frequency Migration (DFM) due to the complex motion of drones is addressed in [65]. This motion is modeled by means of a polynomial function, and different techniques are adopted: the Parametric Symmetric Instantaneous Autocorrelation Function (PSIAF), the Keyston Transform (KT), and the Scaled Fourier Transform (SFT), hence the name of the PSIAF-KT-SFT method. At the end of the processing chain, a CFAR detector ensures the correct threshold setting for the detection problem. Numerical simulations were carried out to evaluate the performance compared to the state-of-the-art, showing that the proposed solution can achieve improved performance. Finally, real experiments on a UAV have shown that range and velocity associated with the target can be accurately identified thanks to the proposed coherent integration.

In [66] the detection of UAVs is addressed in the context of non-Rayleigh clutter, specifically, distributed as a Weibull. By applying a detector based on CA-CFAR, the presence of the target is detected and then identified in range and speed through the 2D FFT map. A further contribution of this work is the time–frequency processing for the visualization of the micro-Doppler signature, which highlights how such a feature is more peculiar of targets like UAVs compared, for example, to a moving person or a helicopter.

In [67] a persistent X-band FMCW radar is considered, which constantly illuminates the area under observation. The radar system consists of a transmitting antenna and eight receiving antennas, all in microstrip technology. Given the number of antennas, it is possible to perform 3D processing for target detection, based on 2D FFT and beamforming. To detect the presence of a target in this case, a two-dimensional CA-CFAR was used that acts in both domains (range and Doppler), so obtaining two distinct thresholds: the lowest is chosen for the actual detection. Any detection that exceeds the resolution cell is also traced back to the single cell by calculating its centroid. In this way it is possible to detect and track a small UAV up to about 3 km away, both in the case of simple flight and in the case of more complex maneuvers. Finally, the authors made further assessments regarding the RCS distribution of these targets, highlighting a certain similarity with the Swerling 1 target model.

In [68] a self-constructed Digital Array Radar (DAR) using COTS components is proposed, in which the receiving array is built by means of Software Defined Radio (SDR). Once the FMCW signal is received, it is processed through matched filter and digital beamformer. To detect the possible presence of a target, a CFAR approach is also used in this case, specifically the OS-CFAR—which as discussed is more robust than CA-CFAR in the presence of interferers near the CUT. Results have highlighted that it is possible to detect small UAVs with such a radar operating in the S-band up to 1.68 km. The main characteristics of the above methods in terms of specific type of radar adopted and possible CFAR property of the proposed algorithms are summarized in Table 3.

## 4. Literature on Drone Verification and Classification

**Figure 10:**
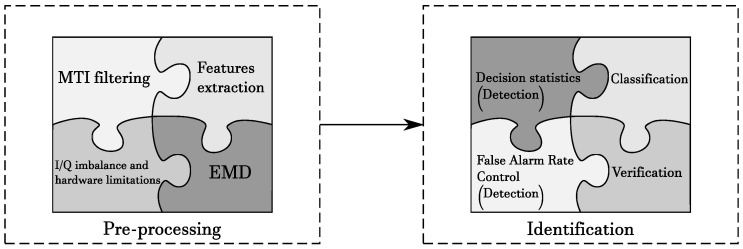
Jigsaw scheme of radar processing for target identification.

A possible scheme for the process of radar-based drone identification is summarized in Figure 10. It is composed of two macro phases, each consisting of sub-elements that are optional depending on the specific objective of the investigation. The components of the first pre-processing phase have been described in Section 2; the remainder of the paper will discuss the components involved in the verification and classification phases, by reviewing the relevant literature.

Most papers have focused on the verification and classification task, thus assuming the initial detection phase has already been accomplished. The most popular solutions concern training and use of machine learning algorithms, neural networks, deep learning, etc. Visual approaches based on image processing or pattern recognition can also be a valid solution, though less popular than the previous ones. For example, the visualization of the spectrogram generates an image that contains a series of information that allows one to extract useful features about the target.

**Figure 11:**
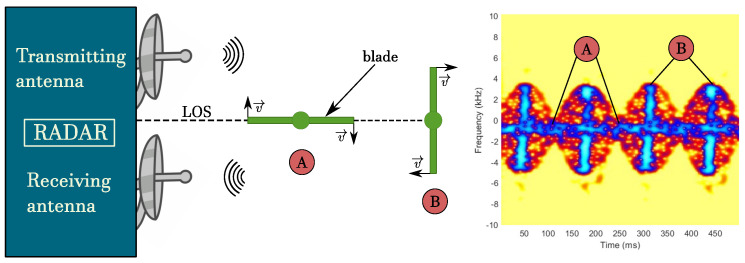
Micro-Doppler modulation of drone propellers.

In all such approaches, the micro-Doppler signature is considered as a key feature characterizing the presence of a drone, to be evaluated through a time–frequency representation. In Figure 11, a schematic representation of the processing flow is given so as to better understand how micro-Doppler can be used for both verification and classification. As it can be seen, the spectrogram highlights some relevant features such as the number of blades and their length. Consequently, it can be exploited to classify the dimension hence the specific type of the observed target. Moreover, this analysis is helpful to distinguish drones from birds, which, as already highlighted, are one of the most common targets that can likely produce a false alarm. Obviously the effectiveness of micro-Doppler for verification or classification is strictly dependent on the quality of the available hardware, and anyway on the size or number of propellers and rotors. In fact, by increasing the number of rotating parts the spectrogram shows a more confused micro-Doppler modulation hence the classification becomes more difficult.

Drone classification is often based on machine learning algorithms, which are trained through considerable amounts of data that brings their performance to very high levels of accuracy. Since a number of reviews already covered this literature, for example, [69,70], in the following, only some representative approaches will be discussed, which can be used to verify and/or classify a target that has been detected through one of the approaches discussed in the previous section.

Classification of different categories of drones can be obtained by performing the STFT to obtain spectrograms, and then extract some features to feed a classifier. In [59], after the detection phase they addressed the classification problem. Firstly they perform a micro-Doppler analysis using different techniques such as CVD, spectrogram, cesptogram, and complex CVD, for subsequent features representation. The PCA is used for feature extraction, so as to obtain a preliminary classification by SVM. In a second phase, the current time segment and a number of previous segments are processed to declare the final decision (presence or absence of a target). The null class (absence of a target) includes possible nuisance targets that can be found in an urban scenario, such as vehicles, bicycles, walking people, etc. In [60] the STFT is computed, after detection phase, to obtain the spectrogram, the cepstogram, and finally the CVD, from which features for classification are extracted. The aim was to emphasize the possible presence of payloads carried by small drones in flying and hovering mode. Finally a Convolutional Neural Network (CNN), specifically AlexNet, is trained to effectively classify. In [71] data are collected from two radar sensors (in the K and X bands) and the classification task is performed both individually and for their combination. After a preliminary Principal Component Analysis (PCA)-based reduction step, a Support Vector Machine (SVM) classifier is used to discriminate among the different categories of drones. It emerged that the classification accuracy of the combination of the two radar sensors is higher than for the case of a single radar sensor.

In [72] the problem of distinguishing drones from birds is addressed, following previous work in [73,74,75]. Specifically, the category of drones is considered in alternative to the category of flying objects in which birds and other flying objects fall. The measurements campaign involved different types of small UAVs and birds. After the computation of STFTs to evaluate the Time Velocity Diagram (TVD), six physical features defined by [76] are extracted for classification purposes. A boosting classifier (LogitBoost) is finally used to distinguish between drones and birds (target detection) and types of drones (target classification) with good results compared to SVM.

In [77] the classification problem is addressed by trying to correctly discriminate mini-UAVs from both birds and stationary rotors that are not UAV. Once the STFT is computed, a classification method based on the extraction of six entropy measures is adopted. These entropies are extracted from the IMFs after EMD, and each of them contains different information regarding the signal under analysis. An X-band CW radar was used to perform the experiment. Among the investigated entropy measures, Shannon, spectral, and log energy were the most effective ones; such features were finally used in a nonlinear SVM classifier.

In [78] the characteristic micro-Doppler properties of three drones and four birds of different size have been analyzed with the aim of creating an extensive dataset of drones and birds signatures useful for classification. Two typologies of radar were used for the data acquisition: a K-band (24 GHz) and a W-band (94 GHz). Higher frequencies lead to better Doppler resolution in a shorter time, which in turn could be very useful to make some features more evident. By using STFT, it has been shown that the micro-Doppler characteristics of drones and birds differ significantly and therefore can be used as metrics to distinguish between the two categories. Different STFT window lengths (namely different resolutions) have been used to inspect the features of different types of flying drones (e.g., number of rotors). Moreover, authors observed that the micro-Doppler return from the W-band radar experienced a higher SNR compared to the one obtained from the K-band radar.

In [79] drones are classified using the micro-Doppler signature in UHF, which represents a low-frequency with respect to the usual frequency ranges, hence an additional challenge. By combining STFT, spectral subtraction, EMD and PCA, some drone features are extracted and fed into four different classifiers: SVM, Naive Bayes (NB), K-Nearest Neighbor (KNN) and Random Forest. Two typologies of drones were used to evaluate the classification performance. The obtained results confirmed that the micro-Doppler can effectively guarantee good performance for these purposes.

In [80], a follow-up of [81], a COTS FMCW radar system is adopted for UAV classification, assuming the target is persistent in the scene. The micro-Doppler signature and 13 different features were extracted from IMFs and fed to a classifier based on total error rate minimization. Different typologies of objects have been used to evaluate the classification performance, such as cars, moving people, bicycles and many different types of drones. Finally, the TER-based classifier is compared against linear and nonlinear SVM, obtaining more accurate results.

In [61] the focus is put on the classification of targets carrying different payloads; in fact, although there is a slight change in RCS between drones with and without payload, there is no significant indicator for this classification task. An S-band multistatic FMCW radar system (NetRAD) has been used to collect the data; then, Doppler and bandwidth centroid of the micro-Doppler signatures are processed through STFT and Singular Value Decomposition (SVD) to extract some features that can characterize micro-drone hovering and flying with different payloads. Three classifiers are used for classification: NB, a diagonal-linear variant of the Discriminant Analysis Classifier (DAC), and the Random Forest classifier. Results showed that the classification accuracy improves when separate classifiers are used to process the data from different radar nodes, with respect to a single classification performed in a centralized process. Satisfactory classification accuracy has been obtained using the SVD for moving micro-drones carrying payloads, whereas the centroid-based approach turned out to be more advantageous in the presence of hovering micro-drones. Table 4 provides a summary of the features and type of classifiers used in the above-reviewed papers on drone classification.

## 5. Conclusions

Automatic identification of small UAVs is a timely and important problem: the widespread of drones is indeed enabling unprecedented applications but, at the same time, new threats are arising linked to their possible misuse (e.g., drug smuggling, terrorist attacks, espionage). There is still a gap in current surveillance systems to cope with this problem, and the main challenges are linked to the detection, possible verification, and classification tasks. Modern surveillance systems composed of a network of spatially-distributed sensors is the most promising approach to ensure a full-coverage of the monitored area, and to take advantage of the pros of different technologies while coping with their individual cons. This review has focused more specifically on radar sensors, which is a key technology also due to its low cost and capability to work at relatively long distances, as well as strong robustness to illumination and weather conditions. The reviewed literature, as a whole, indicates that a mix of different solutions is necessary to face this challenging problem; at the same time, more sophisticated signal processing algorithms are still needed to improve the detection and classification performance.

## Figures and Tables

**Table 1:** Comparison among drone surveillance technologies.

Technology	Approach	Pros and Cons
Video	One or more cameras to perform identification exploiting drone motion	Very accurate at short distances Good for verification and classification Sensitive to low ambient light, variable illumination, and occlusions Hard to distinguish drones from other small flying objects at long range
Audio	Sound generated by flying drones exploited to perform DOA-based identification	Useful to distinguish drones from birds based on acoustic signatures Good for verification Effective only at very short distances
RF (passive)	Downlink video stream or EM scattering of opportunistic RF signals	Very effective for drone detection Possible localization of remote pilot Increased false alarm rate due to interference (crowded ISM band) Ineffective for autonomous drones
Radar (RF active)	Backscattering of RF signal exploited to perform Doppler-based tracking and delay-based identification	Robust to weather and illumination conditions Very effective for drone detection and classification Probability of detection highly dependent on radar cross section
LIDAR (laser scanner)	Similar to radar, but backscattering of laser light is exploited	Sensitive to bad visibility due to weather, smog, or direct sunlight Very effective for drone detection Basic classification is possible based on target size, but drones and birds indistinguishable

**Table 2:** CFAR algorithms and their operating scenarios.

	Environment			
	Homogeneous	Interfering Targets	Clutter Boundaries	Interfering Targets and Clutter Boundaries
CA	✓			
GOCA			✓	
SOCA		✓		
CS		✓		
TM		✓	✓	✓
OS		✓	✓	✓
GOOS		✓	✓	✓
GOCS		✓	✓	✓

**Table 3:** Main characteristics of the reviewed drone detection methods.

Paper	Radar Type	Frequency Band	CFAR
[59]	CW	K-band	✓
[60]	multistatic pulsed	S-band	✓
[62]	FMCW	K-band	✗
[63]	FMCW	W-band	✓
[64]	multistatic pulsed	S-band	✓
[65]	FMCW	X-band	✗
[66]	FMCW	K-band	✓
[67]	FMCW	X-band	✓
[68]	FMCW	S-band	✓

**Table 4:** Type of radar, operating frequency, features and specific classifiers used in the reviewed drone classification algorithms.

Paper	Radar Type	Frequency Band	Features	Classifier
[59]	CW	K-band	Micro-Doppler signature	SVM
[60]	multistatic pulsed	S-band	Micro-Doppler signature	CNN (AlexNet)
[61]	FMCW	S-band	Micro-Doppler signature	NB, DAC, Random Forest
[71]	CW	X and K bands	Micro-Doppler signature	SVM
[72]	CW	X-band	6 physical features from [76]	LogitBoost
[77]	CW	X-band	6 entropy measures from IMF	SVM
[78]	FMCW	K and W bands	Micro-Doppler signature	not specified
[79]	CW	UHF	Micro-Doppler signature	SVM, KNN, NB, Random Forest
[80]	FMCW	X-band	Micro-Doppler signature and 13 IMF features	TER

## Abbreviations

The following abbreviations are used in this manuscript:

CA-CFAR	Cell Averaging CFAR
CFAR	Constant False Alarm Rate
CNN	Convolutional Neural Network
COTS	Commercial-Off-The-Shelf
CVD	Cadence Velocity Diagram
CW	Continuous Wave
DAR	Digital Array Radar
DFM	Doppler Frequency Migration
DOA	Direction Of Arrival
EMD	Empirical Mode Decomposition
FFT	Fast Fourier Transform
FIR	Finite Impulse Response
FMCW	Frequency Modulated Continuous Wave
GPS	Global Positioning System
IID	Independent and Identically Distributed
IMF	Intrinsic Mode Function
KNN	K-Nearest Neighbor
LFMCW	Linear Frequency Modulated Continuous Wave
MTI	Moving Target Indicator
NB	Naive Bayes
OS-CFAR	Order Statistics CFAR
PCA	Principal Component Analysis
PD	Probability of Detection
PDF	Probability density Function
PFA	Probability of False Alarm
RCS	Radar Cross Section
RF	Radio Frequency
RM	Range Migration
SDR	Software Defined Radio
SNR	Signal-to-Noise-Ratio
SOCA-CFAR	Smallest of CA-CFAR
STFT	Short Time Fourier Transform
SVD	Singular Value Decomposition
SVM	Support Vector Machine
UAV	Unmanned Aerial Vehicle
